# Marine Sponge *Aaptos suberitoides* Extract Improves Antiproliferation and Apoptosis of Breast Cancer Cells without Cytotoxicity to Normal Cells In Vitro

**DOI:** 10.3390/ph15121575

**Published:** 2022-12-16

**Authors:** Jun-Ping Shiau, Min-Yu Lee, Jen-Yang Tang, Hsin Huang, Zheng-Yu Lin, Jui-Hsin Su, Ming-Feng Hou, Yuan-Bin Cheng, Hsueh-Wei Chang

**Affiliations:** 1 Division of Breast Oncology and Surgery, Department of Surgery, Kaohsiung Medical University Hospital, Kaohsiung Medical University, Kaohsiung 80708, Taiwan; 2 Graduate Institute of Medicine, College of Medicine, Kaohsiung Medical University, Kaohsiung 80708, Taiwan; 3 School of Post-Baccalaureate Medicine, Kaohsiung Medical University, Kaohsiung 80708, Taiwan; 4 Department of Radiation Oncology, Kaohsiung Medical University Hospital, Kaoshiung Medical University, Kaohsiung 80708, Taiwan; 5 Department of Marine Biotechnology and Resources, National Sun Yat-sen University, Kaohsiung 80424, Taiwan; 6 Department of Biomedical Science and Environmental Biology, College of Life Science, Kaohsiung Medical University, Kaohsiung 80708, Taiwan; 7 Center for Cancer Research, Kaohsiung Medical University, Kaohsiung 80708, Taiwan

**Keywords:** *Aaptos suberitoides*, marine sponges, natural product, breast cancer, oxidative stress

## Abstract

The anticancer effects and mechanisms of marine sponge *Aaptos suberitoides* were rarely assessed, especially for methanol extract of *A. suberitoides* (MEAS) to breast cancer cells. This study evaluated the differential suppression effects of proliferation by MEAS between breast cancer and normal cells. MEAS demonstrated more antiproliferation impact on breast cancer cells than normal cells, indicating oxidative stress-dependent preferential antiproliferation effects on breast cancer cells but not for normal cells. Several oxidative stress-associated responses were highly induced by MEAS in breast cancer cells but not normal cells, including the generations of cellular and mitochondrial oxidative stress as well as the depletion of mitochondrial membrane potential. MEAS downregulated cellular antioxidants such as glutathione, partly contributing to the upregulation of oxidative stress in breast cancer cells. This preferential oxidative stress generation is accompanied by more DNA damage (γH2AX and 8-hydroxy-2-deoxyguanosine) in breast cancer cells than in normal cells. *N*-acetylcysteine reverted these MEAS-triggered responses. In conclusion, MEAS is a potential natural product for treating breast cancer cells with the characteristics of preferential antiproliferation function without cytotoxicity to normal cells in vitro.

## 1. Introduction

Breast cancer accounts for 30% of female cancer and is the leading cause of women’s cancer death [1]. It increases by 0.5% per year. Three major subtypes characterize most breast cancer cells, i.e., estrogen receptor (ER), progesterone receptor (PR), and human epidermal growth factor receptor 2 (HER2) [2]. Some 10–15% of breast cancers belong to triple-negative breast cancer (TNBC), i.e., no ER/PR/HER2 [3], which is hard to cure using target therapy. Identifying more anticancer drugs against breast cancer cells is still necessary, particularly for TNBC.

Marine sponges are rich in diverse natural products [4,5,6,7,8] for curing cancer treatments [6]. For example, 70% aqueous ethanol extract of *Grayella cyathophora* inhibits the proliferation of colon and breast cancer cells [9]. Methanol extract of *Crambe* displays antiproliferation against pancreatic cancer cells [10]. Marine sponge *Lipastrotethya* sp. extract suppresses colon cancer cell proliferation [11]. Ethyl acetate extract from the marine sponge *Stylissa carteri* was reported to inhibit the proliferation of breast cancer cells [12]. Sponge-derived natural products such as hemimycalin C, D, E, and manzamine A show antiproliferation against colon cancer cells [13]. Accordingly, several extracts and bioactive components of marine sponges exhibit anticancer effects.

*Aaptos suberitoides* (*A. suberitoides*) is a marine sponge harvesting in Indonesian waters [14], and it was also found on Orchid Island, Taiwan, in the present study. Recently, the ethanol extract of *A. suberitoides* was reported to inhibit the proliferation and migration of breast cancer cells [15]. However, this study did not examine the detailed anticancer mechanism of ethanol extract of *A. suberitoides*.

The crude extract of natural products containing several bioactive compounds is expected to exhibit multi-targeting effects against cancer cells with low cytotoxicity to normal cells [16]. Sponges include diverse compounds [17]. Hence, sponge extracts have an improving impact on the antiproliferation of cancer cells. Accordingly, the anticancer effects of *A. suberitoides* crude extract warrant a detailed investigation of breast cancer cells.

The present investigation assesses the in vitro antiproliferation effects mechanisms of methanol extract of *A. suberitoides* (MEAS) to breast cancer cells.

## 2. Results

### 2.1. HPLC Analysis of MEAS and Aaptamine

**Figure 1:**
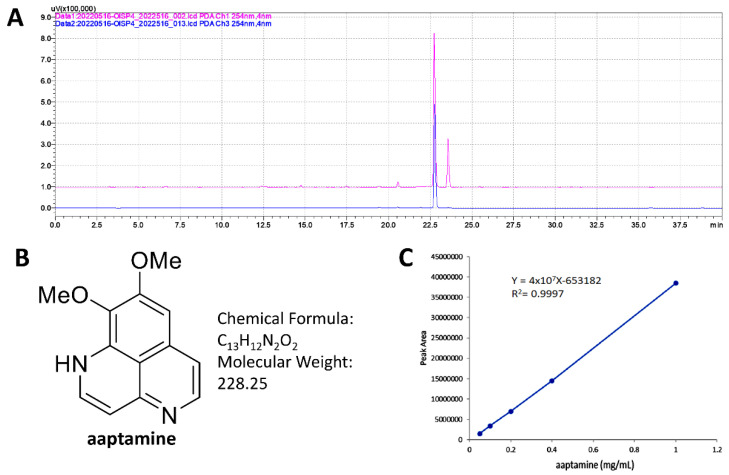
(**A**) The HPLC fingerprint profiles of MEAS and the main product of MEAS (aaptamine) were shown in red and blue, respectively. (**B**) Structure of aaptamine. (**C**) The calibration curve of aaptamine (*n* = 3).

In Figure 1A, the HPLC fingerprint profiles of MEAS (red line) and the main product of MEAS (aaptamine) (blue line) at 254 nm were shown. Aaptamine [18] (Figure 1B) was found to appear at 22.730 min, which overlapped the major peak of MEAS. The NMR spectrum of aaptamine is provided in Supplementary Figure S1. The linear equations (Y = 4 × 10^7^ X − 653182, R^2^ = 0.9997) of aaptamine was deduced by the HPLC peak area in different concentration (Figure 1C). As a result, aaptamine accounts for 15.3% of MEAS.

### 2.2. Antiproliferation of MEAS-Treated Breast Cancer and Normal Cells

**Figure 2:**
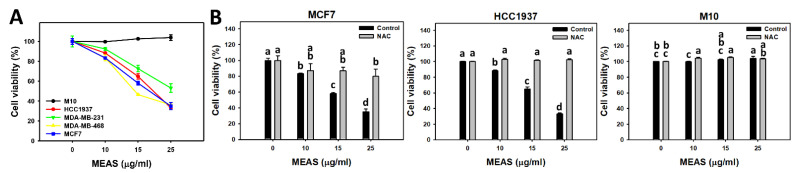
MEAS decreased the viability of several kinds of breast cancer cells. (**A**) Cell viability of MEAS at 24 h MTS assay. Except for normal breast cells (M10), others were TNBC and non-TNBC cells. (**B**) Cell viability of NAC/MEAS at 24 h MTS assay. NAC/MEAS represents NAC pretreatment (10 mM, 1 h) and MEAS posttreatment for 24 h. Data = mean ± SD (*n* = 3). Statistical software assigned low-case letters to each treatment. When the letters of different treatments overlapped, the results were significant (*p* < 0.05). In the example of Figure 2B (MCF7 cells), MEAS treatments at 0, 10, 15, and 25 μg/mL labeled with “a, b, c, and d” differ significantly, determined by non-overlapping conditions. MEAS and NAC/MEAS treatments of MCF7 cells at 10 μg/mL MEAS labeled with “b and ab” show a non-significant difference because they overlapped with “b”, while at 15 μg/mL MEAS labeled with “c and ab” show a significant difference.

In 24 h MTS viability assay, MEAS reduced the cell viability of breast cancer cells (HCC1937, MDA-MB-231, MDA-MB-468, and MCF7) (Figure 2A). For comparison, MEAS showed high viability of normal cells (H184B5F5/M10; M10) [19,20,21] in tested concentrations of MEAS compared to breast cancer cells. These results revealed the preferential antiproliferation character of MEAS on breast cancer cells showing minor changes to normal cells.

MCF7 and HCC1937 cells with high sensitivity to MEAS were chosen to perform the following experiments. The ROS suppressor NAC mitigated the MEAS-caused inhibitory effects of proliferation against breast cancer cells (Figure 2B). Accordingly, oxidative stress is involved in the antiproliferation of MEAS.

### 2.3. Cell Cycle Status of MEAS-Treated Breast Cancer and Normal Cells

**Figure 3:**
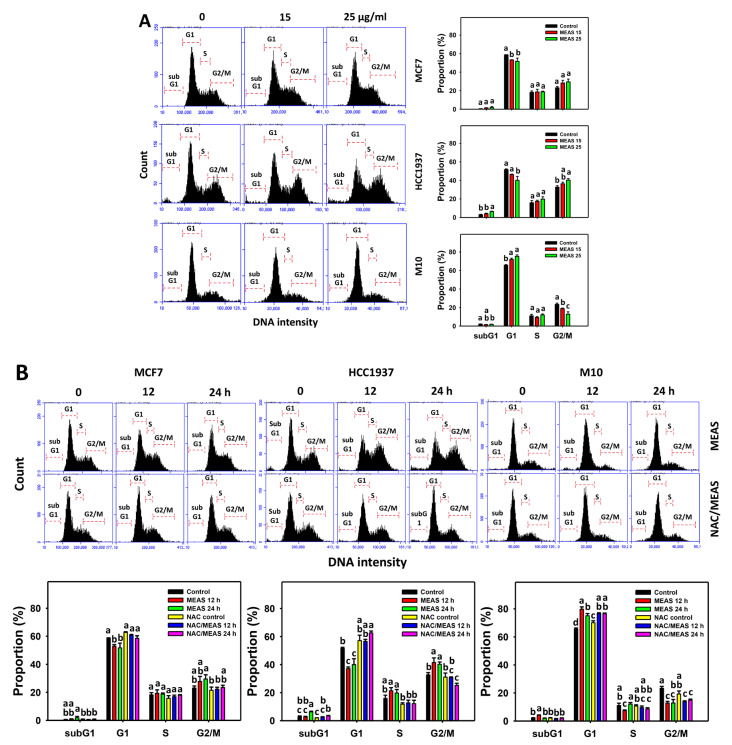
MEAS causes cell cycle redistribution of breast cancer cells. (**A**) Cell cycle assay of MEAS. Except for normal breast cells (M10), others were TNBC and non-TNBC cells. Flow cytometry was performed after 24 h drug treatment. (**B**) Cell cycle assay of NAC/MEAS. NAC/MEAS represents NAC pretreatment (10 mM, 1 h) and MEAS posttreatment for 0, 12, and 24 h. Data = mean ± SD (*n* = 3). Statistical software assigned low-case letters to each treatment. When the letters of different treatments overlapped, the results were significant (*p* < 0.05).

In 24 h 7-amino actinomycin D (7AAD) assay, the subG1 phase (%) of breast cancer cells (MCF7 and HCC1937) and normal cells (M10) [19,20,21] were deficient (Figure 3A). The G1 phase (%) was decreased, and the G2/M phase (%) was increased in breast cancer cells. In comparison, the changes in G1 and G2/M phase (%) in M10 cells were opposite to breast cancer cells.

Additionally, NAC mitigated the MEAS-caused G1-inducible and G2/M-suppressing effects against breast cancer cells at 12 h treatment for MCF7 cells and at 12 and 24 h for HCC1937 cells (Figure 3B). Accordingly, oxidative stress is involved in the cell cycle disturbance effects of MEAS on breast cancer cells.

### 2.4. Annexin V Status of MEAS-Treated Breast Cancer and Normal Cells

**Figure 4:**
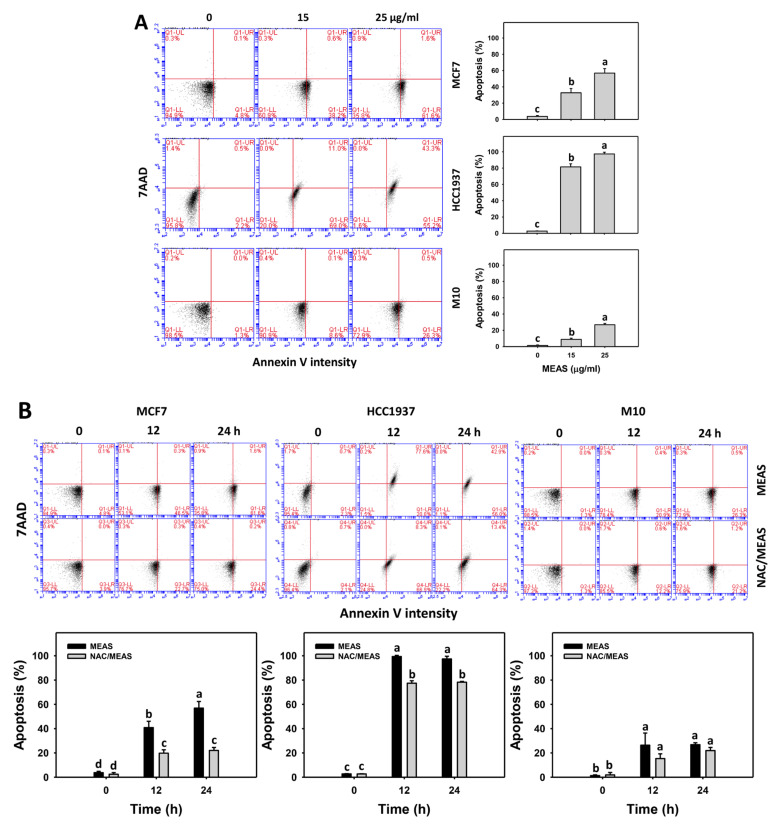
MEAS increased the annexin V intensity of breast cancer cells. (**A**) Annexin V-apoptosis assay of MEAS. Except for normal breast cells (M10), others were TNBC and non-TNBC cells. Flow cytometry was performed after 24 h drug treatment. Annexin V (+) (%), such as annexin V (+)/7AAD (+, −), accounts for apoptosis (%). (**B**) Annexin V-apoptosis assay of NAC/MEAS. NAC/MEAS represents NAC pretreatment (10 mM, 1 h) and MEAS posttreatment for 0, 12, and 24 h. Data = mean ± SD (*n* = 3). Statistical software assigned low-case letters to each treatment.

Annexin V/7ADD assay was adopted for validating apoptosis. MEAS increased the annexin V intensity (+) (%) of breast cancer cells (MCF7 and HCC1937) (Figure 4A). For comparison, MEAS showed high annexin V intensity (+) (%) of breast cancer cells in tested concentrations of MEAS compared to normal cells (M10). These results revealed the preferential apoptosis (annexin V) of MEAS on breast cancer cells showing minor changes to normal cells.

Additionally, NAC mitigated the MEAS-caused annexin V-detected apoptosis against breast cancer cells (Figure 4B). Accordingly, oxidative stress is involved in the apoptosis (annexin V) of MEAS.

### 2.5. Caspase Status of MEAS-Treated Breast Cancer and Normal Cells

**Figure 5:**
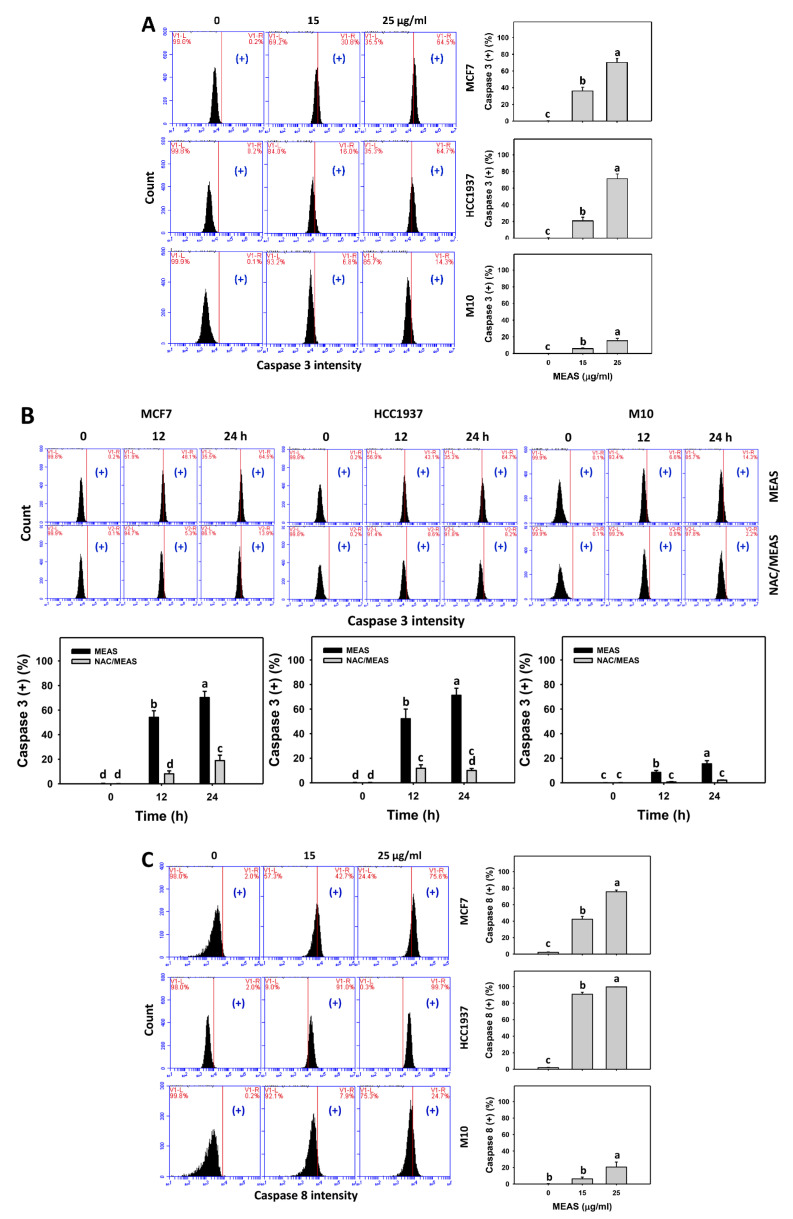

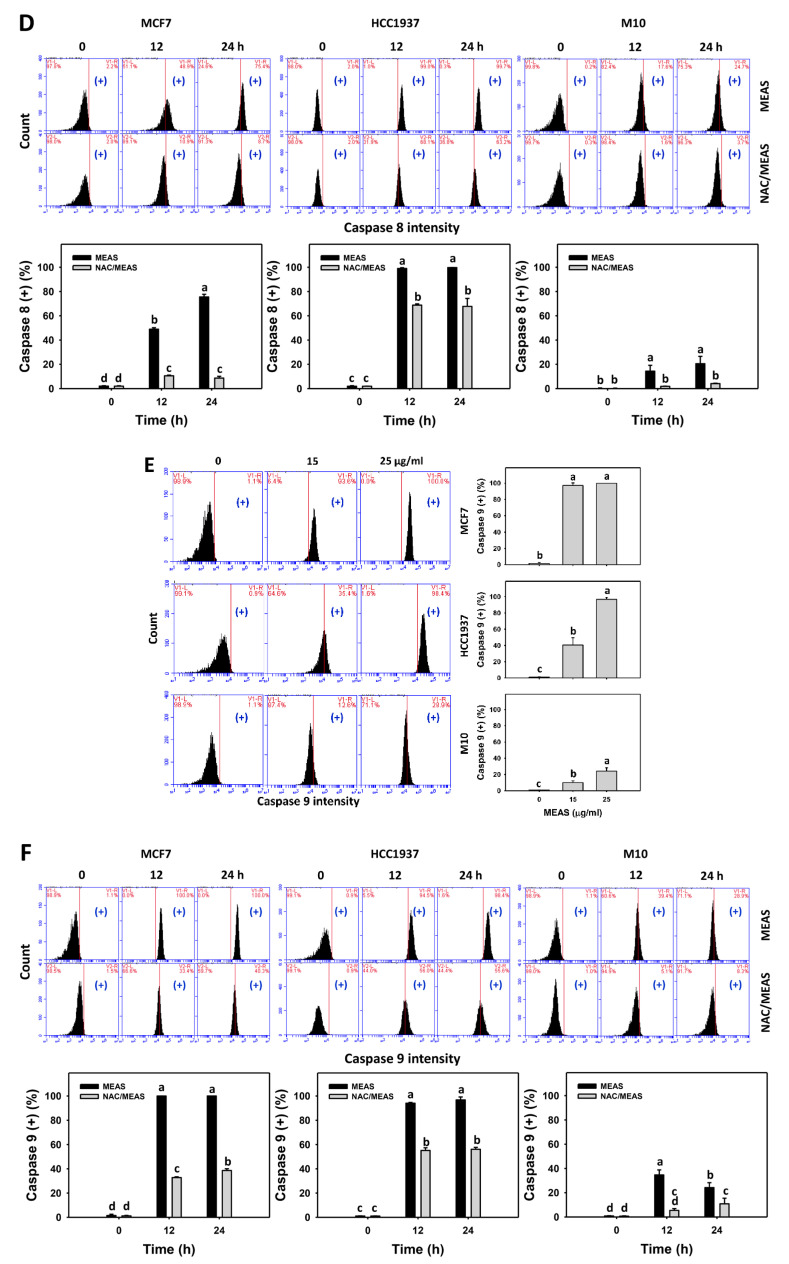
MEAS increased activation of Caspases 3, 8, and 9 of breast cancer cells. (**A**,**C**,**E**) Caspases 3, 8, and 9 activation assays of MEAS. Except for normal breast cells (M10), others were TNBC and non-TNBC cells. Flow cytometry was performed after 24 h drug treatment. (+), inserted within each panel, indicates the caspases 3, 8, and 9 (+) intensity. (**B**,**D**,**F**) Caspases 3, 8, and 9 activation assays of NAC/MEAS. NAC/MEAS represents NAC pretreatment (10 mM, 1 h) and MEAS posttreatment for 0, 12, and 24 h. Data = mean ± SD (*n* = 3). Statistical software assigned low-case letters to each treatment.

Caspase signaling activations such as Caspases 3, 8, and 9 were used to assess apoptosis. MEAS increased the Caspases 3, 8, and 9 intensities (+) (%) of breast cancer cells (MCF7 and HCC1937) (Figure 5A,C,E). For comparison, MEAS showed high Cas 3, 8, and 9 intensities (+) (%) of breast cancer cells in tested concentrations of MEAS compared to normal cells. These results revealed the preferential apoptosis (Caspases 3, 8, and 9) of MEAS on breast cancer cells showing minor changes to normal cells (M10).

Additionally, NAC mitigated the MEAS-caused Caspases 3, 8, and 9-detected apoptosis against breast cancer cells (Figure 5B,D,F). Accordingly, oxidative stress is involved in the apoptosis (Caspases 3, 8, and 9) of MEAS.

### 2.6. Reactive Oxygen Species (ROS) and Mitochondrial Superoxide (MitoSOX) Status of MEAS-Treated Breast Cancer and Normal Cells

**Figure 6:**
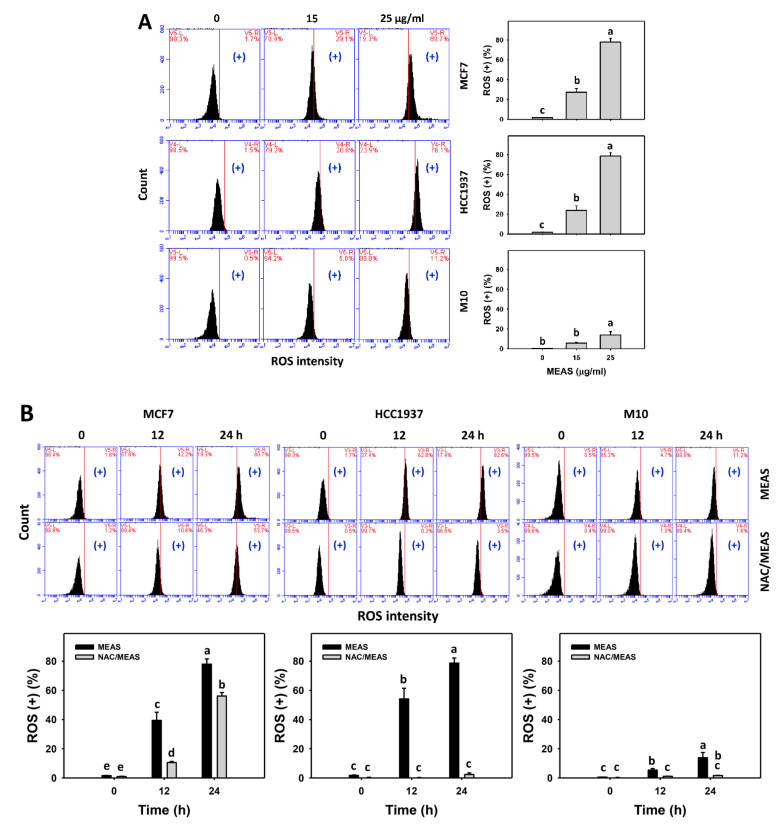

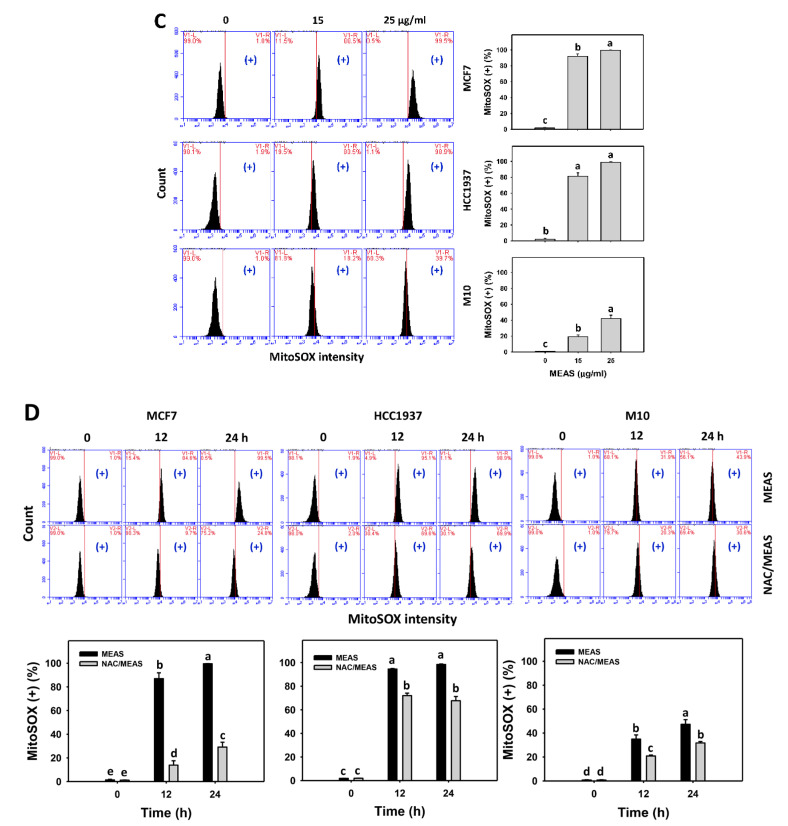
MEAS increased ROS and MitoSOX intensities of breast cancer cells. (**A**,**C**) ROS and MitoSOX assays of MEAS. Except for normal breast cells (M10), others were TNBC and non-TNBC cells. Flow cytometry was performed after 24 h drug treatment. (+), inserted within each panel, indicates the ROS and MitoSOX (+) intensity. (**B**,**D**) ROS and MitoSOX assays of NAC/MEAS. NAC/MEAS represents NAC pretreatment (10 mM, 1 h) and MEAS posttreatment for 0, 12, and 24 h. Data = mean ± SD (*n* = 3). Statistical software assigned low-case letters to each treatment.

MEAS increased oxidative stress, such as ROS and MitoSOX intensities (+) (%) of breast cancer cells (MCF7 and HCC1937) (Figure 6A,C). For comparison, MEAS showed high ROS and MitoSOX intensities (+) (%) of breast cancer cells in tested concentrations of MEAS compared to normal cells. These results revealed the preferential oxidative stress (ROS and MitoSOX) of MEAS on breast cancer cells showing minor changes to normal cells (M10).

Additionally, NAC mitigated the MEAS-caused ROS and MitoSOX against breast cancer cells (Figure 6B,D). Accordingly, oxidative stress is involved in the oxidative stress (ROS and MitoSOX) of MEAS.

### 2.7. Mitochondrial Membrane Potential (MMP) Status of MEAS-Treated Breast Cancer and Normal Cells

**Figure 7:**
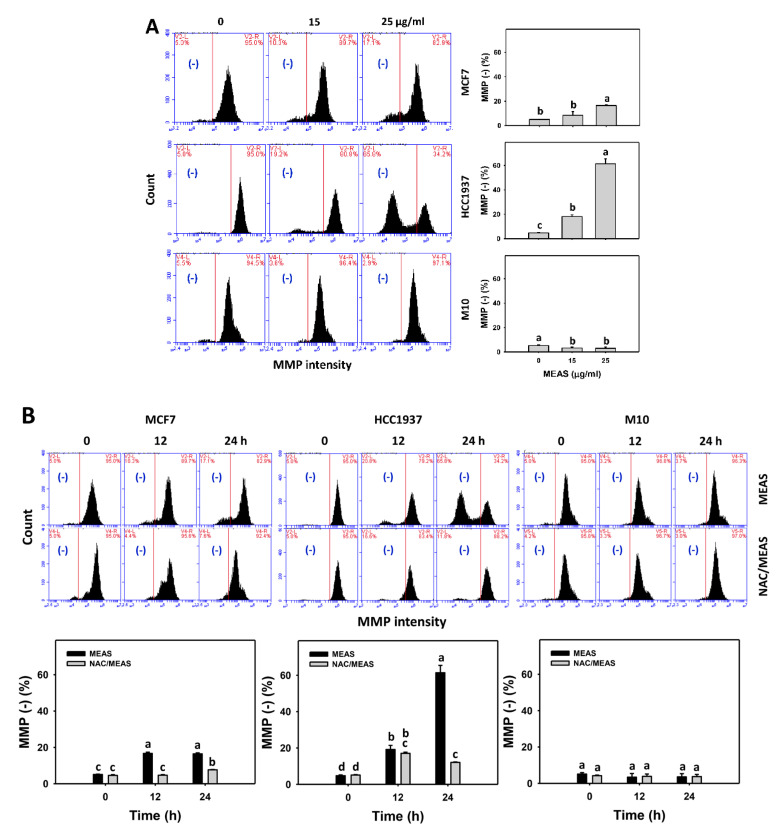
MEAS increased MMP (−) intensities of breast cancer cells. (**A**) MMP assay of MEAS. Except for normal breast cells (M10), others were TNBC and non-TNBC cells. Flow cytometry was performed after 24 h drug treatment. (−), inserted within each panel, indicates the MMP (−) intensity. (**B**) MMP assay of NAC/MEAS. NAC/MEAS represents NAC pretreatment (10 mM, 1 h) and MEAS posttreatment for 0, 12, and 24 h. Data = mean ± SD (*n* = 3). Statistical software assigned low-case letters to each treatment.

MEAS increased the oxidative stress, such as MMP intensity (−) (%) of breast cancer cells (MCF7 and HCC1937) (Figure 7A). For comparison, MEAS showed high MMP intensity (−) (%) of breast cancer cells in tested concentrations of MEAS compared to normal cells. These results revealed the preferential oxidative stress (MMP depletion) of MEAS on breast cancer cells showing minor changes to normal cells (M10).

Additionally, NAC mitigated the MEAS-caused MMP depletion against breast cancer cells (Figure 7B). Accordingly, oxidative stress is involved in the oxidative stress (MMP depletion) of MEAS.

### 2.8. Glutathione (GSH) Status of MEAS-Treated Breast Cancer and Normal Cells

**Figure 8:**
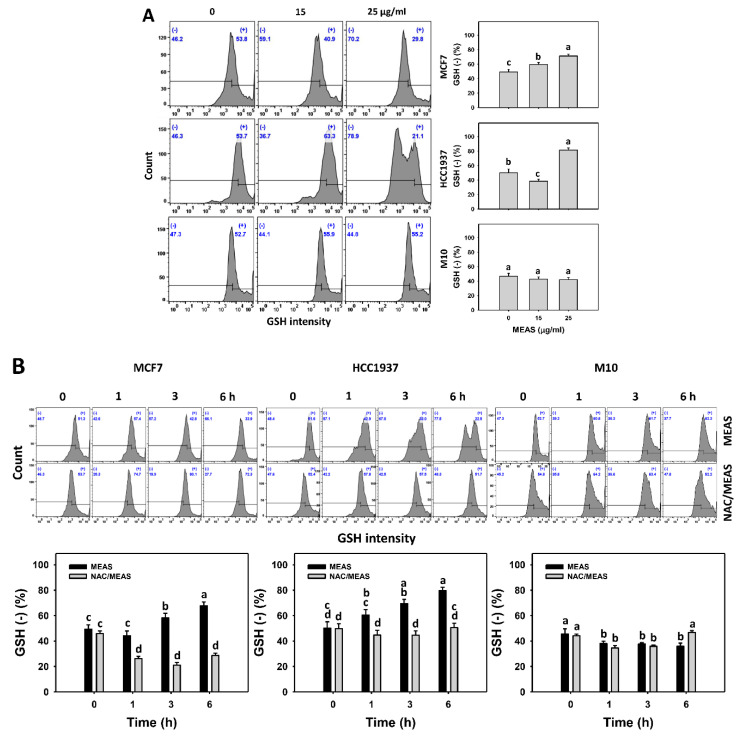
MEAS increased GSH (−) intensities of breast cancer cells. (**A**) GSH assay of MEAS. Except for normal breast cells (M10), others were TNBC and non-TNBC cells. Flow cytometry was performed after 6 h drug treatment. (−), inserted within each panel, indicates the GSH (−) intensity. (**B**) GSH assay of NAC/MEAS. NAC/MEAS represents NAC pretreatment (10 mM, 1 h) and MEAS posttreatment for 0, 1, 3, and 6 h. Data = mean ± SD (*n* = 3). Statistical software assigned low-case letters to each treatment.

The contribution of cellular antioxidants such as GSH in elevating oxidative stress of breast cancer cells treated with MEAS was evaluated. MEAS increased the oxidative stress, such as GSH intensity (−) (%) of breast cancer cells (MCF7 and HCC1937) (Figure 8A). For comparison, MEAS showed high GSH intensity (−) (%) of breast cancer cells in tested concentrations of MEAS compared to normal cells. These results revealed the preferential oxidative stress (GSH depletion) of MEAS on breast cancer cells showing minor changes to normal cells (M10).

Additionally, NAC mitigated the MEAS-caused GSH depletion against breast cancer cells (Figure 8B). Accordingly, oxidative stress is involved in the oxidative stress (GSH depletion) of MEAS.

### 2.9. DNA Damages Status of MEAS-Treated Breast Cancer and Normal Cells

**Figure 9:**
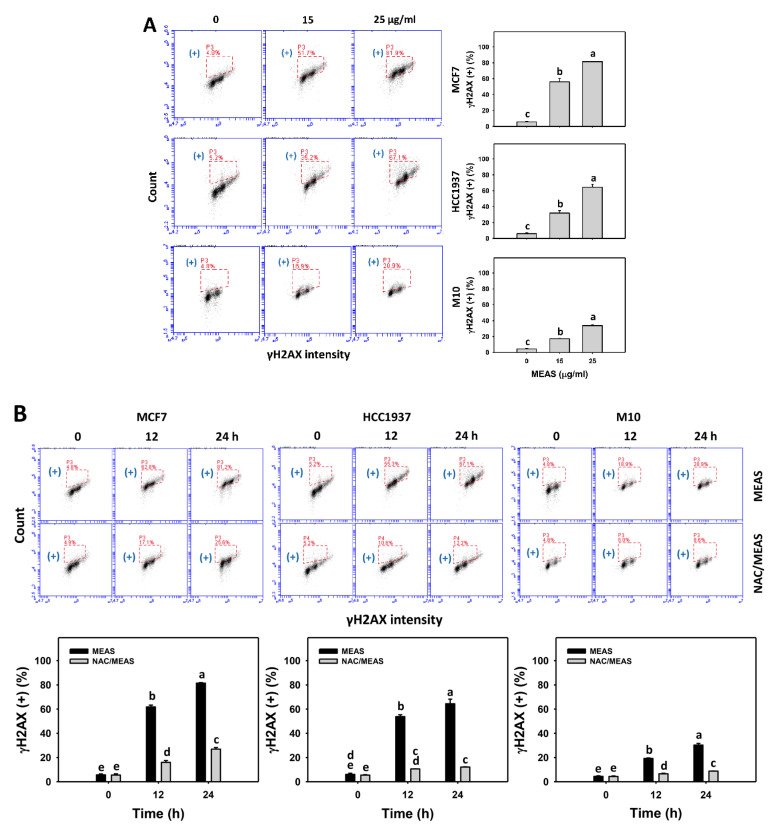
MEAS increased γH2AX intensity of breast cancer cells. (**A**) γH2AX assay of MEAS. Except for normal breast cells (M10), others were TNBC and non-TNBC cells. Flow cytometry was performed after 24 h drug treatment. (+), inserted within each panel, indicates the γH2AX (+) intensity. (**B**) γH2AX assay of NAC/MEAS. NAC/MEAS represents NAC pretreatment (10 mM, 1 h) and MEAS posttreatment for 0, 12, and 24 h. Data = mean ± SD (*n* = 3). Statistical software assigned low-case letters to each treatment.

**Figure 10:**
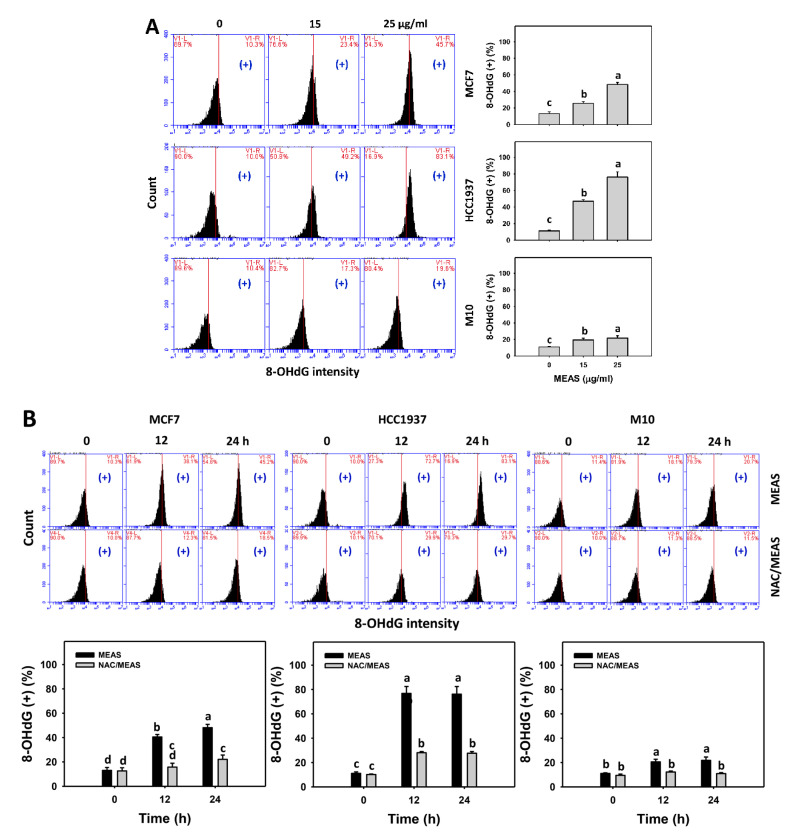
MEAS increased the 8-OHdG intensity of breast cancer cells. (**A**) 8-OHdG assay of MEAS. Except for normal breast cells (M10), others were TNBC and non-TNBC cells. Flow cytometry was performed after 24 h drug treatment. (+), inserted within each panel, indicates the 8-OHdG (+) intensity. (**B**) 8-OHdG assay of NAC/MEAS. NAC/MEAS represents NAC pretreatment (10 mM, 1 h) and MEAS posttreatment for 0, 12, and 24 h. Data = mean ± SD (*n* = 3). Statistical software assigned low-case letters to each treatment.

MEAS increased the DNA damage such as γH2AX and 8-hydroxy-2-Deoxyguanosine (8-OHdG) intensities (+) (%) of breast cancer cells (MCF7 and HCC1937) (Figure 9A and Figure 10A). For comparison, MEAS showed high γH2AX and 8-OHdG intensities (+) (%) of breast cancer cells in tested concentrations of MEAS compared to normal cells. These results revealed the preferential DNA damage (γH2AX and 8-OHdG) of MEAS on breast cancer cells showing minor changes to normal cells (M10).

Additionally, NAC mitigated the MEAS-caused γH2AX and 8-OHdG against breast cancer cells (Figure 9B and Figure 10B). Accordingly, oxidative stress is involved in the damage (γH2AX and 8-OHdG) of MEAS.

## 3. Discussion

Marine sponges are abundant in natural products for anticancer drug discovery [4,5,6,7,8,10,22,23,24]. The anti-breast cancer effects of ethanol extract of *A. suberitoides* were reported, such as antiproliferation and antimigration [15], but it did not provide a detailed investigation of the anticancer mechanism. The present study examined the antiproliferation effects and mechanism of methanol extract of *A. suberitoides* (MEAS) between breast cancer and normal cells.

Several marine sponge extracts showed anticancer effects. The ethanol extract of *Aaptos suberitoides* shows an IC_50_ value of 12.0 μg/mL to breast cancer cells (HCC-1954) at 72 h MTT assay [15]. 70% aqueous ethanol extracts of *Grayella cyathophora* and *Negombata magnifica* show IC_50_ values of 2.14 and 1.09 μg/mL to colon cancer cells (Coca-2) at 72 h MTT assay [9]. A 70% ethanol extract of *Dysidea avara* shows IC_50_ values of 11.51, 5.11, and 17.54 μg/mL to the breast (MCF7), cervical (HeLa), and colon (HCT116) cancer cells at 48 h MTT assay [25]. Notably, these studies did not consider the drug’s safety for normal cells. They did not investigate the cytotoxicity of normal cells.

In contrast, IC_50_ values of MEAS at 24 h MTS assay are 17.81, 19.19, 24.41, and 14.23 μg/mL in breast cancer cells (MCF7, HCC1937, MDA-MB-231, and MDA-MB-468, respectively). Both TNBC and non-TNBC cells were sensitive to MEAS. Moreover, the viability of normal cells (M10) [19,20,21] is higher than breast cancer cells following MEAS treatment, suggesting the preferential antiproliferation effects on breast cancer cells. This antiproliferation is modulated by oxidative stress as validated by NAC pretreatment (Figure 2B).

The oxidative stress involvement in MEAS treatment of breast cancer cells was validated by ROS and MitoSOX generation and MMP depletion (Figure 6 and Figure 7). These oxidative stresses were higher in breast cancer cells than in normal cells, indicating MEAS triggers the preferential induction of oxidative stress in breast cancer cells.

The redox status is controlled by the balance between cellular antioxidants and prooxidants [26]. When the levels of prooxidants are higher than that of oxidants, cells suffer from oxidative stress. The suppression of cellular antioxidants is one of the reasons for generating oxidative stress [27]. For example, alantolactone, an *Inula helenium*-derived natural product, causes oxidative stress by depleting GSH levels of glioblastoma cells [28]. The brown algae-derived fucoidan triggers GSH downregulation to induce oxidative stress in oral cancer cells [29]. Similarly, MEAS promoted GSH depletion in breast cancer cells to a greater extent than in normal cells (Figure 8). Moreover, this MEAS-induced GSH depletion was reversed by NAC pretreatment, a precursor for GSH biosynthesis. Consequently, MEAS stimulates the preferential induction of oxidative stress against breast cancer cells.

Moreover, several marine sponge extracts showed apoptosis-inducible function in cancer cells [25,30,31,32]. For example, ethanol extract of *Dysidea avara* shows apoptosis of cervical and leukemia cancer cells by annexin V detection [25]. The *N*-hexane extract of *Hyrtios erectus* causes subG1 accumulation and activates caspase 3 and caspase 9 in breast cancer cells [33]. For comparison, the subG1 population is few after MEAS treatments for breast cancer and normal cells (Figure 3). However, the subG1 accumulation is not an essential apoptotic response [34]. In some cases, no prominent subG1 peaks are observed in drug-induced apoptosis, depending on the exposure time of drug treatment. For the example of (-)-anonaine treatment to lung cancer cells, the subG1 peaks are very low at 24 and 48 h but high at 72 h [35]. Notably, MEAS triggered apoptosis (annexin V and caspase 3) (Figure 4 and Figure 5) and turned on both extrinsic and intrinsic caspases (caspases 8 and 9) (Figure 5). MEAS promoted more apoptosis in breast cancer cells than in normal cells. This character of preferential apoptosis may attribute to its impact on preferential oxidative stress. Additionally, the MEAS-induced oxidative stress in breast cancer cells promotes a greater extent of DNA damage, such as γH2AX and 8-OHdG, than in normal cells (Figure 9 and Figure 10).

Finally, the impacts of oxidative stress acting on the MEAS-induced cell cycle, oxidative stress, and DNA damage were validated by NAC pretreatment. Therefore, MEAS exerts an oxidative stress-associated mechanism for preferential antiproliferation against breast cancer cells in vitro.

## 4. Materials and Methods

### 4.1. Sample Collection and Identification

The animal material of *Aaptos suberitoides* was obtained from Orchid Island, Taitung County, Taiwan, in April 2011. The voucher specimen (OISP-4) was given, and the specimen was stored at the Department of Marine Biotechnology and Resources, National Sun Yat-sen University, Kaohsiung, Taiwan. The sponge was identified by co-author Dr. Jui-Hsin Su using the DNA sequence and the sponge spicule morphology.

### 4.2. Sample Preparation

The animal material (2.3 kg) was extracted by ethanol three times, and the crude extract was partitioned by EtOAc and H_2_O to give an EtOAc soluble portion. This portion was further partitioned by H_2_O/methanol/hexanes (1:3:4). After removing the solvents, the methanol extract portion (5.8 g) of *A. suberitoides* (MEAS) was used for further chromatographic analysis and pharmacological experiments.

### 4.3. Isolation of Aaptamine

The MEAS was subjected to vacuum liquid chromatography (VLC) eluting with hexanes/CH_2_Cl_2_/MeOH (increasing polarity), and ten fractions (Fr. 1 to Fr. 10) were obtained. Fr. 5 (610.6 mg) was purified by a silica gel open column eluting with CH_2_Cl_2_/MeOH (10:1 to 0:1) to afford three subfractions (Fr. 5-1 to Fr. 5-3). Fr. 5-2 (577.2 mg) was further purified by CC on LH-20 eluting with 100% MeOH to give aaptamine (492.3 mg) [36].

### 4.4. HPLC Analysis of A. suberitoides

Separation by high-pressure liquid chromatography (HPLC) was accomplished on a Shimadzu LC-40D solvent delivery module equipped with the Phenomenex Luna 5 µ C18(2) 100A analytical column. The Shimadzu SPD-M40A photodiode array detector and CTO-40S column oven were selected for analysis. The chromatography methods were listed below: Solution A: 0.1% trifluoroacetic acid_(aq)_; solution B: MeCN; flow rate: 1.0 mL/min; 0 min: 1% solution B, 0–35 min: 1% to 40% solution B, 35–40 min: 40%-100% solution B.

### 4.5. Cell Cultures and Inhibitors

ATCC breast cancer cell lines, such as MCF7 (Luminal A type), and several TNBC cell lines, such as MDA-MB-468 [37], MDA-MB-231, and HCC1937 [38], were included and maintained in DMEM/F12 (3:2) (Gibco, Grand Island, NY, USA) with 10% fetal bovine serum and P/S antibiotics. A non-malignant normal breast epithelial cell line (H184B5F5/M10) [19,20,21] was used as control, which was purchased from Bioresource Collection and Research Center (Hsinchu, Taiwan) and maintained in alpha medium with 10% bovine serum (Gibco, Grand Island, NY) and P/S antibiotics. *N*-acetylcysteine (NAC) (Sigma-Aldrich, St. Louis, MO, USA) [39,40,41,42] is a glutathione precursor that was commonly used as ROS inhibitor. Under 10 mM for 1 h pretreatment, cells were post-treated with MEAS as indicated in figure legends.

### 4.6. Cell Viability

Cell viability was detected by Promega’s MTS kit (Madison, WI, USA). Cells were seeded and incubated overnight. Subsequently, cells were used for drug treatment. Finally, MTS reagents were mixed with medium for a 1 h reaction and read by an ELISA reader at 490 nm [29].

### 4.7. Cell Cycle

Fixed cells were incubated with 1 μg/mL of 7AAD (Biotium; Hayward, CA, USA) [43,44]. The DNA content of 7AAD-positive cells was then detected by the Accuri C6 flow cytometer (BD Biosciences, Franklin Lakes, NJ, USA).

### 4.8. Apoptosis

Annexin V/7AAD double-staining [45,46] and caspases 3, 8, and 9 activation assays [47] were used to detect apoptosis status. Annexin V-FITC/7AAD (1:1000/1 μg/mL) [48] (Strong Biotech; Taipei, Taiwan) was added to cells for 1 h incubation, and their fluorescence intensities were detected by Accuri C6 flow cytometer. The cell spots for annexin V (+)/7AAD (+ or −) intensity were counted as apoptosis (+) cells.

Moreover, the activities of caspases 3, 8, and 9 were detected by OncoImmunin’s specific peptides (Gaithersburg, MD, USA) according to the manufacturer’s instructions [47,49]. When caspases 3, 8, and 9 are activated, these peptides generate fluorescence and are detected by the Accuri C6 flow cytometer.

### 4.9. Oxidative Stress

Several oxidative stress-related indicators, such as ROS, MitoSOX, MMP, and GSH, were tested. They were respectively detected by 2′,7′-dichlorodihydrofluorescein diacetate (H_2_DCFDA) (Sigma-Aldrich) [41,50], MitoSOX™ Red [51] (Thermo Fisher Scientific, Carlsbad, CA), DiOC_2_(3) (Invitrogen, San Diego, CA, USA) [52], and 5-chloromethylfluorescein diacetate (CMF-DA) (Thermo Fisher Scientific, Carlsbad, CA, USA) (5 μM, 20 min) [29], according to manufacturer’s instructions. In response to these oxidative stresses, the Accuri C6 cytometer detected the generating fluorescence of ROS, MitoSOX, and MMP, while the Guava easyCyte flow cytometer (Luminex, TX, USA) detected the generating fluorescence of GSH.

### 4.10. DNA Damages

Several DNA damage indicators, such as γH2AX and 8-OHdG, were detected. Cells need to process with 75% ethanol fixation before antibody reactions. Except for the extra step for 7AAD (5 μg/mL, 30 min) incubation, γH2AX and 8-OHdG [29] were detected by specific antibodies such as γH2AX [53,54] (Santa Cruz Biotechnology; Santa Cruz, CA, USA)/Alexa Fluor 488-secondary antibody (Cell Signaling Technology, Danvers, MA, USA) and FITC-8-OHdG antibody (Santa Cruz Biotechnology), respectively. Finally, these fluorescence intensities were detected by the Accuri C6 flow cytometer.

### 4.11. Statistical Analysis

JMP software (SAS Institute Inc., Cary, NC, USA) was used to determine significance. It provides connecting letters for each treatment. When the connecting letters were not overlapped, treatments differed significantly. Examples for determining significance were given in the figure legend.

## 5. Conclusions

The present study validated the oxidative stress-dependent antiproliferation effects of MEAS in breast cancer cells. MEAS promotes more antiproliferation of breast cancer cells than normal cells. The anti-breast cancer effects of MEAS exert dysregulation of the cell cycle and oxidative stress in breast cancer cells. This abnormal oxidative stress sequentially induces higher apoptosis and DNA damage in breast cancer cells than in normal cells. Therefore, MEAS is a potential marine natural product for breast cancer treatment.

## Data Availability

Data are contained within the article.

## Supplementary Materials

The following supporting information can be downloaded at: https://www.mdpi.com/article/10.3390/ph15121575/s1, Figure S1: ^1^H and ^13^C NMR spectra of aaptamine.
