# Supplementary figures and images for: Marine Sponge Aaptos suberitoides Extract Improves Antiproliferation and Apoptosis of Breast Cancer Cells without Cytotoxicity to Normal Cells In Vitro
Source: Pharmaceuticals (Basel). 2022 Dec 16;15(12):1575. doi: 10.3390/ph15121575 (PMC9783771; doi:10.3390/ph15121575)

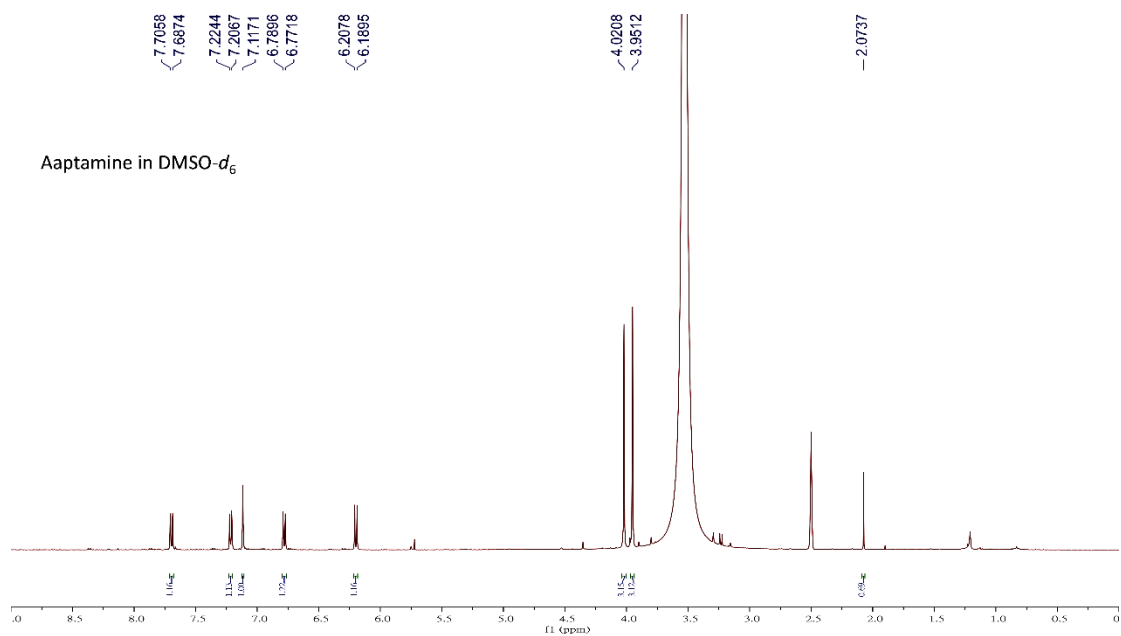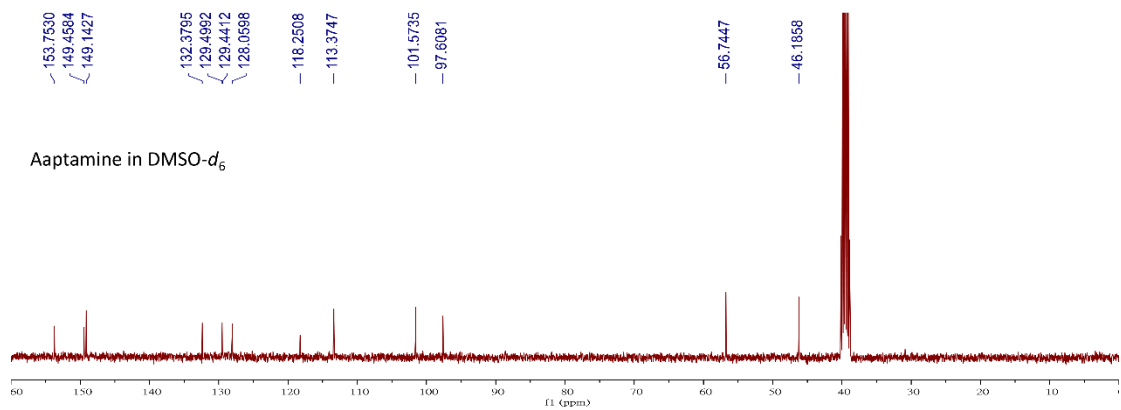

**Supplementary Figure S1.**  $^1\text{H}$  and  $^{13}\text{C}$  NMR spectra of aptamine.

Supplement: Supplementary file 1 [file pharmaceuticals-15-01575-s001.zip › pharmaceuticals-2084659-supplementary.pdf]
